# Utilizing Theory of Planned Behaviour to increase intention to participate in hepatitis C treatment therapy among Methadone maintenance therapy clients (MMT) in Malaysia: A cluster randomised control trial

**DOI:** 10.1371/journal.pone.0324718

**Published:** 2025-05-22

**Authors:** Mohd Hafiidz Baharudin, Siti Aisah Mokhtar, Ahmad Zaid Fattah Azman, Ahmad Iqmer Nashriq Mohd Nazan

**Affiliations:** 1 Department of Community Health, Faculty of Medicine and Health Sciences, Universiti Putra Malaysia, Serdang, Selangor, Malaysia; 2 Ministry of Health, Kuala Muda District Health Office, Kedah, Malaysia; Centers for Disease Control and Prevention, UNITED STATES OF AMERICA

## Abstract

**Introduction:**

The World Health Organization (WHO) has targeted eliminating viral hepatitis as a public health problem by 2030. Thus, high-risk groups such as Methadone Maintenance Therapy (MMT) clients should be targeted for hepatitis C health intervention to increase intention for hepatitis C infection treatment. This study aims to evaluate the effectiveness of theory-based hepatitis C health education and learning module (HEAL) in increasing intention to participate in hepatitis C treatment among MMT clients in Malacca.

**Methods:**

A single-blinded cluster randomised control trial was conducted among selected MMT clients from government health clinics in Malacca from July 2023 to February 2024. The clinics involved was randomly allocated into intervention and control groups. The intervention group received HEAL module developed based on the Theory of Planned Behavioural while the control group attended clinic session as usual. Generalized Estimating Equation (GEE) analysis was used for statistical analysis.

**Results:**

GEE shows that there is significant interaction between time and group for both intention for hepatitis C treatment and knowledge on hepatitis C. Intervention group at immediately post intervention follow up had higher intention for hepatitis C treatment as compared to control group (β = 0.56, 95% CI = 0.12, 1.01, p-value < 0.01). However, there were no significant different in intention for hepatitis C treatment between the two group at 3-month post intervention follow up. Furthermore, intervention group had significant higher knowledge at immediately post intervention (β = 8.85, 95% CI = 7.35, 10.36, p-value < 0.001) and at 3-month post intervention (β = 5.25, 95% CI = 3.76, 6.75, p-value < 0.001) as compared to the control group. However, the knowledge level among the intervention group reduces at 3-month post intervention follow up as compared to knowledge level at immediately post intervention follow up.

**Conclusion:**

HEAL module which utilizes motion video and group discussion was effective in increasing MMT client’s intention for hepatitis C treatment and knowledge about hepatitis C. However, future research should focus on finding effective strategies to ensure retention of the outcomes of HEAL module intervention over time.

## Introduction

### Background

Hepatitis C had been recognized by the World Health Organization as major public health problem [1]. From 58 million people living with hepatitis C, only around 15.2 million knew their hepatitis C status [2]. Yearly, around 1.5 million new infection and around 290 000 mortalities related to hepatitis C were recorded [2]. Furthermore, as mortality from other communicable disease such as human immunodeficiency virus (HIV), tuberculosis and malaria were declining,mortality related to hepatitis C was recorded to continue to increase by 22% since year 2000 and was predicted to increase yearly further if not intervene [1]. Fortunately, in recent decades, effective curable treatment using the Direct acting antiviral therapy (DAA) was discovered. As compared to old treatment using Interferon regime, treatment by DAA offered a shorter duration of treatment with fix dose combination pills that comes with milder tolerable side effect and higher post-treatment sustained virologic response (SVR) rate (80% to 90%) along with lower relapse rate [3–6]. However, treatment coverage for hepatitis C using this new regime is still very poor. It was recorded that only 9.5 million people with hepatitis C had been treated with DAA between 2015–2019 as compared to 58 million estimated people with hepatitis C [2].

Methadone maintenance therapy (MMT) program is a program introduced as a harm reduction program to prevent and control HIV and other blood borne infection resulting from needle sharing among people who injecting drugs (PWID). It was found to be effective in reducing injecting behaviors among PWID in several studies [7,8]. However, several studies conducted in America, China and several European countries have described high prevalence of hepatitis C infection among their MMT clients respondents ranging between 26% to 96% [9–14]. Similarly, a study in Malaysia has reported a high hepatitis C prevalence of 64.6% among MMT clients [15]. Despite these, only 13.7% to 48% of hepatitis C infected MMT clients had undergo hepatitis C treatment which reflect the low hepatitis C treatment uptake among this populations [10–14]. Fortunately, several studies have also proven that MMT programs have essential roles in linking high-risk populations to hepatitis C screening and treatment [9,14,16]. Therefore, MMT program play important roles in providing hepatitis C infection education, screening, and treatment referrals.

Several studies have identified factors affecting the intention and uptake of hepatitis C treatment among MMT clients. A lack of knowledge about hepatitis C and the cost of treatment are significant barriers to initiating care [17]. Additionally, concerns about side effects from older treatment regimens impact the willingness to seek treatment [18–21]. One study found that most respondents were unaware of newer treatments, which have fewer major side effects than the old interferon regimen [14]. Prior attendance at hepatitis C educational activities and higher knowledge levels are significantly associated with the willingness to initiate treatment [22]. Furthermore, MMT clients with concerned friends or partners and those receiving information about hepatitis C treatment from peers are more likely to engage in treatment [14].

Hepatitis C infections were found to be highly prevalent among MMT clients in Malaysia which accounted for 64.6% of the MMT clients [15]. Data have shown that a third of death among MMT clients were related to hepatitis C infection [15]. Unfortunately in term of treatment, Malaysia were still in the process of expending the hepatitis C treatment, the generic DAA regimes were only started to be introduce and available at selected MOH facilities where limited numbers of patients can be treated in stages [23,24]. Currently, it was estimate that treatment were only available for around 23000 patient as compared to around 380000 person diagnosed with chronic hepatitis C [24,25]. However, in achieving Hepatitis C elimination target, Malaysia is working hard to upscale the hepatitis C treatment at all health facilities and the treatment was expected to be available at all facilities within the next few years. This is evident by the launching of the national hepatitis C program that aims to decentralize the treatment of uncomplicated hepatitis C to government health clinics [26]. Despite this effort, it was observed that the awareness and knowledge regarding hepatitis C and the fact that its curable were still very low among MMT clients [27,28]. Moreover, some healthcare workers involve in hepatitis C care on MMT clients find it difficult to convince clients for hepatitis C screening and treatment which reflect the low hepatitis C treatment coverage of 1.4% reported in 2018 [27–29]. This reflect that the intention of MMT clients to received hepatitis C treatment upon diagnosis were still low and the needs for a program or module to prepare them and increase their intention for treatment as the government scaled up the hepatitis C treatment in Malaysia.

It is important to ensure the potential receiving population were informed and ready to accept the treatment voluntarily when the treatment program have been expended. The motivation, effort and willingness to go for treatment is well suited to the term behavioural intention which is a part of construct of Theory of Planned Behaviour (TPB) developed by [30]. The behavioural theory seeks to explain human behaviour by analysing the antecedent and consequences present in the individual’s environment and the learned associations the individual acquire through previous experience [31]. Thus, for a more structured and to ensure high success of intervention module developed in achieving it outcome, this study incorporate TPB in the development of the hepatitis C health education intervention module.

This study seeks to develop, implement, and evaluate the effectiveness of a newly developed theory-based hepatitis C health education intervention module (HEAL) in increasing intention for hepatitis C treatment among MMT clients. It also aims to evaluate the effectiveness of the module in improving respondent knowledge on hepatitis C infection and treatment. Currently, from literature review, no study was found to have evaluated the effectiveness of application of TPB-based hepatitis C health education intervention module in increasing the intention for hepatitis C treatment among MMT client. The use of behavioural theory and multimodal intervention methods in developing and delivering this module is expected to significantly increase awareness of hepatitis C and enhance respondents’ intention to seek treatment. This module is paramount in achieving these outcomes because it applies proven behavioural theories and utilizes an interactive delivery method, which was derived from a systematic review conducted prior to its development. Unlike conventional delivery methods, such as standard health talks, this interactive approach fosters greater engagement and retention of information, leading to a more substantial impact on awareness and treatment intentions. Thus, this study will add to the body of knowledge on the effectiveness of Theory of Planned behaviour-based hepatitis C health education intervention module in increasing the intention for hepatitis C treatment among MMT clients in Malacca.

Malacca, a state in southern Malaysia, is recognized as a key transit area in regional drug trafficking routes between Malaysia and Indonesia [32]. In recent years, the state has experienced an increase in number of individuals undergoing MMT for substance abuse. This growing population of drug users places them at higher risk for hepatitis C transmission. As a result, Malacca serves as an important location to evaluate this intervention, particularly among high-risk groups such as MMT clients.

## Methods

### Population

This two-armed, parallel, single blinded, cluster randomized control trial (CRCT) was conducted among Methadone Maintenance Therapy (MMT) clients attending Methadone clinics at government health clinics in Malacca from July 2023 until February 2024. Malacca was a state in Malaysia located at latitude and longitude coordinates of 2.200844, 102.240143 respectively. In Malaysia, the government Methadone clinics was the main facilities who provide opioids replacement therapy to people who use opioids (PWUO), who is diagnosed with opioids dependence. The cluster that was enrolled into the study includes government Methadone clinics with more than 10 MMT clients and does not taken part in the development of the study instruments. A total of 19 clinics was eligible to be recruited and out of these, 18 clinics were further randomly allocated to enrolled into intervention and control arm of the study.

### Eligibility criteria

For the eligibility criteria at the cluster level, clinics must be government health clinics in Malacca that provide methadone maintenance therapy services and have more than 10 active clients. Clinics that were involved in the development and testing of the study instrument were excluded from the study. At the individual respondent level, participants must be active clients who are 18 years or older and have a hepatitis C status of either non-reactive, unknown, or reactive but treatment naïve. Eligible clients were further interviewed for consent and enrolment in the study. Clients who had been diagnosed with hepatitis C and were ever or currently on treatment were excluded from the study.

### Sampling method

The sequence generation and allocation concealment process was conducted by the main investigator while the implementation of the study which include enrolment of participants, opening the envelop, data collection via questionnaire was performed by trained liaison officer at site in accordance to suggestion by the CONSORT (Consolidated Standard of Reporting Trials) statement [33]. This statement provides a paragraph to ensure transparency and rigour in reporting randomized controlled trials. The CONSORT statement emphasizes proper randomization, allocation concealment, and clear reporting of participant flow to enhance the reliability and validity of trial outcomes.

Malacca had a total of 33 government health clinics. However, 7 health clinics do not have methadone services, and another 7 health clinics does not fulfil the eligibility criteria’s to be recruited into the study. Thus, a total of 18 clinics were randomly selected from 19 clinics who were eligible for recruitment using simple random sampling. The one clinic that was not included into study was reserve for the testing and quality control of the tools of the study prior to the commencement of the study. Random allocation sequence was generated to randomly allocate 9 clusters into intervention group and the remaining 9 into the control group. The permuted block randomization was used to allocate cluster into either the intervention or control group. This method is used to ensure close balanced numbers of clinics being allocated to each arm of the study [33]. In addition, “sequentially numbered, opaque, sealed enveloped” allocation concealment methods were used to ensure that the implementation of random allocation sequence occur without prior knowledge of which clinics will be allocated into the intervention or control group. The randomisation code was released after clinics have been recruited into the study which take place after the baseline measurement have been completed [34]. By doing this, the parties involve during the randomization process was blinded from the group that the clinics were allocated before the unique code being reveal.

Following allocation of cluster, individuals’ respondents was recruited from each clinic. The list of MMT clients based on the registration number from selected clinics was obtained and were screen for eligibility criteria. Those who does not fulfil the eligibility criteria was removed from the list. Subsequently, the cleaned registration number list was arranged in ascending order. Following that each client was assigned number starting from number one to total number of the population. In the next step simple random sampling method was conducted using online random number generator, to recruit equal number of participants (7 participants) from each clinic to obtained fixed number of equal size clusters according to number of sample size required.

The flow chart of study design was presented using the CONSORT 2010 flow diagram format as shown in S1 Fig, which illustrates the phases of the cluster randomised controlled trial, including the recruitment and allocation of cluster and participants.

### Sample size

The initial sample size for this study was calculated using the formula for testing the difference in proportions between two groups, with the primary outcome being the intention to participate in hepatitis C treatment. The calculation was based on findings from previous studies and provided sufficient power to detect significant difference between the intervention and control groups. The estimated sample size was 39 participants per group under an individual randomization design.

However, given the study employed a cluster randomized controlled trials (CRCT) design, an adjusted was necessary to account for clustering effects. Using the formula:


Nc = nk[1−p]/[k−np]

whereby:

Nc = Sample size adjusted for cluster

n = sample size calculated under individual randomisation (39)

k = number of clusters (9)

p = Intra-cluster correlation coefficient (ICC = 0.05)

The adjusted sample size for clustering was 40 participants per arm (intervention and control groups), accounting for the potential intra-cluster correlation among participants.

To further adjust for potential attrition, we referenced studies involving people with substance misuse, where dropout rates have been reported as high as 30% [32,35]. The formula used for adjusting the sample size for attrition was:


Adjusted Sample Size=1−L40

where:

- **L** = expected dropout rate (0.30).

Thus, the adjusted sample size, accounting for 30% dropout was 57 participants per group, resulting in total required sample size of 114 participants (57 participants in both the intervention and control groups).

### Outcomes

The main outcome of interest in this study is the intention for hepatitis C treatment among MMT clients. It refers to the intention of MMT client for hepatitis C treatment if they were ever diagnosed with hepatitis C and are advise for treatment by the attending physician. Respondent will need to grade four items related to intention for hepatitis C treatment on a seven-point bipolar adjective scale adopted from TPB guidelines [36]. The higher the mean of the intention scores reflect higher intention for hepatitis C treatment. The secondary outcome in this study is the knowledge on hepatitis C infection and treatment. It refers to the respondent knowledge score on hepatitis C infection, transmission, prevention, and treatment as adopted from previous study [37]. The higher the knowledge score of the respondents, the better the knowledge of respondent with regards to hepatitis C infection and treatment.

### Study instruments

#### The questionnaire.

The questionnaire for this study is a self-administered questionnaire with 54 questions and was distributed in Malay language. The questionnaire for measuring intention was adopted from a guideline on constructing TPB based questionnaire [36]. Meanwhile, the questionnaire for knowledge on hepatitis C infection and treatment was adopted and adapted from previous study by Arain et al [37]. Prior to the study, the questionnaire undergoes forward and backward translation from English language to Malay language following the report of the ISPOR Task Force for Translation and Cultural Adaptation (TCA) [38]. It also undergoes validity and reliability testing. Questions with content validity ratio (CVR) lower than 0.99 were either eliminated or modified based on the importance of the questions [39]. In addition, the reliability of the instrument was measured using an internal consistency method. In measuring internal consistency of an instrument, alpha value of 0.6 to 0.7 was consider as acceptable for construct with items more than 10 [40]. For construct with item of less than 10, Cronbach alpha of more than 0.5 is considered as acceptable [41]. All the constructs in the questionnaire were able to achieve acceptable value of Cronbach alpha as mentioned. The summary of internal consistency for scale use in this study is summarized in Table 1.

**Table 1:** Cronbach alpha coefficient for each construct of the study.

Scale	Type of scale	No of items in each scale	Cronbach alpha coefficient
Intention for hepatitis C treatment	Seven-point bipolar adjective scale	4	0.929
Knowledge on hepatitis C infection and treatment	Nominal scale	20	0.929

Following data collection. The score for each scale was calculated. For knowledge construct, total knowledge score was calculated over maximum of 20 score. For intention construct, the mean score was calculated over maximum of 7 score. The higher the score indicates more positive response towards hepatitis C infection and treatments.

#### The Intervention Module (HEAL Module).

The intervention module was developed based on Sidek’s module development model [42–44]. It was developed in Malay language based on input from 7 experts which consist of Public Health specialist and Family Medicine Specialist from Universiti Putra Malaysia (UPM) and Malacca State Health Department. The main aim of the module is to increase the intention of hepatitis C treatment among MMT clients. It also aims to increase awareness of MMT clients on hepatitis C infections, its complication and advancement in its treatment. The submodule covered under this module was based on Theory of Planned Behaviour (TPB) which were found to be suitable to address the factors associated with intention for hepatitis C treatment among MMT clients. The activities and sections of the module was developed based on the construct of the theory which include attitude towards hepatitis C infection and treatment, subjective norms to hepatitis C infection and treatment and perceived behavioural control to hepatitis C treatment. In addition, the construct of knowledge towards hepatitis C infection and treatment were include as an additional construct which was found to be important in increasing intention towards hepatitis C treatment.

Prior to development of the module, a need analysis was conducted through questionnaire answering by 11 experts in the field which consist of public health specialist, family medicine specialist and medical officers who involve and in charge of MMT clinics in health clinics in Malacca. The aim of this analysis is to explore the experts view on current situation regarding hepatitis C awareness and intention for treatment as well as the needs for a theory-based module to increase the awareness of hepatitis C infection and intention for hepatitis C treatment among MMT clients. It also aims to identify the important of TPB construct to be include in the intervention module for increasing intention for hepatitis C treatment. For these purposes, the factors related to TPB construct that should be include in designing the intervention module were also explored. Fuzzy Delphi Method (FDM) was used to analyse the agreement of expert on statement regarding the need for intervention module, intention for treatment among MMT clients and important factors for intervention [45]. Following that the objective, content, and method of delivery of submodule was identify and developed by conducting literature review. The summary of objective, content and method of delivery of each submodule is as summarize in Tables 2 and 3.

**Table 2:** Objective according of each submodule of the intervention.

Sub-module Title	Domain	Learning Objective
1: Understanding hepatitis C infections and its treatment	Knowledge	To increase knowledge regarding hepatitis C infection To increase knowledge regarding hepatitis C treatment
2: Enhancing Positive Attitude towards hepatitis C treatment	Attitude	To increase positive attitude towards hepatitis C treatment. To inform regarding medical and on medical benefit of getting treatment. To inform regarding different between old and newer treatment. To inform regarding consequence of not getting treated.
3: Cultivating a Positive Norm and improving perceived behavioural control towards hepatitis C treatment	Subjective Norm and Perceived behavioural control	To inform regarding physician and peers’ expectation of an individual in getting hepatitis C treatment. To share first-hand experience on positive benefit of getting treatment. To address common individual barrier in getting treatment. To increase self-confident in making decision to get treatment.

**Table 3:** The content and method of delivery of the module.

Sub-module Title	Domain	Content of module	Method of delivery	Duration
1: Understanding hepatitis C infections and its treatment	Knowledge	Presentation on hepatitis C covering: Aetiology of hepatitis C Mode of transmission Risk factors. Diagnosis Sign and symptoms. Course of disease Complication Treatment	Video presentation Discussion	25 minutes
2: Enhancing Positive Attitude towards hepatitis C treatment	Attitude	Presentation on: Medical and non-medical benefit of treatment. Effect of untreated chronic hepatitis C Case presentation on an individual with untreated chronic hepatitis C. Case presentation on an individual with treated chronic hepatitis C.	Video presentation Discussion	25 minutes
3: Cultivating a Positive Norm and improving perceived behavioural control towards hepatitis C treatment	Subjective Norm and Perceived behavioural control	Presentation on interview and sharing by medical practitioner involve in giving hepatitis C treatment and a patient completed the treatment.	Video presentation Discussion	45 minutes

### Implementation of interventions

Participants who meet the eligibility criteria and consented to participate in the intervention was initially subjected to complete the baseline questionnaire. Following that, an appointment date was given for intervention session. The intervention was conducted at around 1–2 weeks from baseline data collection period. The intervention was conducted in the form of small group (7 respondent in a group) face to face session during the appointment date. The intervention was delivered by a trained field investigators in the form of video presentation for each submodule followed by discussion and questions and answering session. The average duration of the intervention was around 90 minutes. Following module presentation, an immediate post intervention questionnaire was filled in by respondents. The respondent was then given another appointment for questionnaire answering session 3 month after the intervention. The total duration of the intervention and follow up was around 4 months. All sessions were conducted at the clinics in which the respondents received their Methadone services.

Respondent in the control group was also subjected to a baseline questionnaire answering session following recruitment. Clinics that were allocate into control group does not receive any intervention module and was put in wait list. These clinics were encouraged to continue their routine hepatitis C health promotion and health education session using already available health education materials in their clinics such as pamphlets and hands out. Participant in the wait list will receive the intervention module once the study is completed and the module was found to be effective in enhancing the desired outcomes.

### Statistical analysis

IBM SPSS (Version 29.0) was used for data analysis. Data was first explored to evaluate missing data in term of amount, pattern, and type of missing data. Following that, the distribution of continuous data was determined to identify whether they were normally or not normally distributed. For descriptive analysis, continuous data that was normally distributed was presented in the form of mean and standard deviation while for data that was not normally distributed, median, and interquartile range was used to describe the data. Categorical data was described in the form of frequency and percentage. For between group baseline data analysis, the independent sample t-test was used to compare the mean different of continuous and normally distributed data between intervention and control group at baseline. For continuous but not normally distributed data, the Mann-Whitney U test was used to compare between intervention and control group at baseline. The Chi-square test was used to compare frequency different of categorical data between intervention and control groups and for data of 2 by 2 table that contains cell with an expected count less than 5 for more than 20%, the Fisher’s exact test was used. For variable with more than 2 categories Fisher-Freeman Halton Test was used to compare frequency different of categorical data between intervention and control groups.

The Generalized Estimating Equation (GEE) approach was used to compare the differences in intention and knowledge regarding hepatitis C infection and treatment between the intervention and control groups across time points, adjusted to covariates. GEE was chosen for its ability to handle correlated data within clusters, which is particularly important in cluster randomised controlled trials (CRCT) such as this one, where participants were clustered based on the clinics they attended.

Two outcome measures were assessed, which is the intention to participate in hepatitis C treatment and knowledge about hepatitis C. For intention outcome, which is continuous, we applied the Gaussian family of distribution with identity link function to model population-averaged effect over time. Similarly, for the knowledge outcome, treated as a continuous variable, the Gaussian family with identity link was also used.

This study adopted the Intention-to-treat (ITT) analysis strategy during data analysis. This strategy imply that all participants who was randomized are included in the statistical analysis and analysed according to the group they were originally assigned, regardless of what treatment they received [46]. This allow for accurate (unbiased) conclusions regarding the effectiveness of an intervention and preserves the benefits of randomization, which cannot be assumed when using other methods of analysis [46].

### Ethical consideration

Prior to commencement of data collection and intervention sessions, ethical approval was obtained from Medical Review and Ethics Committee (MREC), Ministry of health Malaysia (NMRR ID-23-00016-XJF (IIR)) as well as approval to conduct study from Malacca state health department. Furthermore, this study was registered under Thai Clinical Trial Registry (TCTR): TCTR20240504001. In addition, written inform consent was obtained from each individual respondent before participating in the study (voluntary participation). All data obtained from the study was handled with strict confidentiality and privacy. Participants have the right to withdraw from participating in this study at any time.

### Deviation from the initial protocol

This study was initially developed and submitted to the ethical board as a randomize control trial (RCT) study. However, upon further discussion with the supervisory committee and after analysing the feasibility of conducting the study after obtaining ethical approval, this study was change to a cluster randomized control trial study mainly to prevent contamination of intervention module from intervention group to the control group which can interfere with results of the study. Hence all of related aspect of the study such as sample size adjustment, eligibility criteria, sampling method, randomization and data analysis was adjusted accordingly to suite cluster randomized control trial study design.

## Results

### Response rate

Participants recruitment commence from July 2023 until October 2023. All 9 clinics from each arm of the study participated at baseline, immediately post intervention and at 3-month post intervention session. However, at individual level, only 119 MMT clients consented to join the study thus giving 94.5% recruitment rate. Out of the 119 respondents recruited, 57 respondents were from the clinics randomized into the intervention groups and 62 respondents were from the clinics randomize into the control groups. Throughout the study, all 119 respondents were able to participate in the baseline data collection session and immediately post intervention data collection session. However, 2 participants in the control group were loss to follow up during the 3-month post intervention data collection session giving the participant retention rate of 98% at the third session.

### Sociodemographic characteristic at baseline (N=119)

Tables 4 and 5 shows the sociodemographic characteristic of the intervention and control group at baseline. There were three sociodemographic characteristic that show statistically significant different which. This includes race (*X*^2^ = 6.73, p < 0.05), history of non-injecting drug use in 6 month (*X*^2^ = 4.45, p < 0.05) and number of previous Hepatitis C education program attended month (*X*^2^ = 8.76, p < 0.05). These significant variables were adjusted in the final model of GEE analysis as covariates.

**Table 4:** Baseline comparison of sociodemographic characteristics for continuous variable (n = 119).

Characteristics	Intervention (N = 57)		Control (N = 62)		Independent t test		
	Mean	SD	Mean	SD	Mean Different	t Statistic	p Value
Age (years)	47.18	9.99	48.84	10.59	-1.66	-0.880^c^	0.38
Income (RM)	1000.00^a^	850.00^b^	1500.00^a^	975.00^b^	–	–	0.40^d^
MMT Dose (mg/day)	45.00^a^	30.00^b^	45.00^a^	32.50^b^	–	–	0.56^d^
Duration of MMT treatment (years)	6.50^a^	8.00^b^	9.00^a^	7.00^b^	–	–	0.18^d^

Note: (

^a^)- Median, (

^b^) – IQR, (

^c^) – P value for Levene test is > 0.05 thus equal variance assume t statistic was used, (

^d^) – Mann-Whitney U test.

**Table 5:** Baseline comparison of sociodemographic characteristics for categorical variable (n = 119).

Characteristics	Full Sample (n)	Full Sample (%)	Intervention		Control		Chi Square test		
			n	%	n	%	*X* ^2^	d.f.	p value
**Gender**							0.01	1	0.93
Male	115	96.6	55	47.8	60	52.2			
Female	4	3.4	2	50.0	2	50.0			
**Race**							6.73^a^	–	**0.03** ^a^ ^,^ ^*^
Malay	106	89.1	55	51.9	51	48.1			
Chinese	9	7.6	1	11.1	8	88.9			
Indian	3	2.5	1	33.3	2	66.7			
Others	1	0.8	0	0.0	1	100.0			
**Race (Recode)**									
Malay	106	89.1	55	51.9	51	48.1	6.18	1	**0.01** ^*^
Non-Malay	13	10.9	2	15.4	11	84.6			
**Marital Status**							0.91^a^	–	0.86^a^
Single	40	33.6	18	45.0	22	55.0			
Married	57	47.9	29	50.9	28	49.1			
Divorce	6	5.1	2	33.3	4	66.7			
Widow/Widower	16	13.4	8	50.0	8	50.0			
**Occupational Status**							0.85^a^	–	0.69^a^
Have permanent Job	48	40.3	21	43.8	27	56.2			
No permanent Job	54	45.4	28	51.9	26	48.1			
Not working	15	12.6	8	53.3	7	46.7			
**Education**							3.55^a^	–	0.66^a^
No formal education	3	2.5	1	33.3	2	66.7			
Primary School	10	8.4	4	40.0	6	60.0			
Form 1–3	50	42.0	21	42.0	29	58.0			
Form 4–5	47	39.5	25	53.2	22	46.8			
Form 6	2	1.7	1	50.0	1	50.0			
Tertiary Education	7	5.9	5	71.4	2	2.6			
**Smoking Cigarettes**							1.09	1	0.38
Yes	106	89.1	49	46.2	57	53.7			
No	13	10.9	8	61.5	5	38.5			
**SmokingVape/e-Cigarettes**							3.82	1	0.05
Yes	19	16.0	13	68.4	6	31.6			
No	100	84.0	44	44.0	56	56.0			
**Ever Injecting Drug use**							1.03	1	0.31
Yes	47	39.5	25	53.2	22	46.8			
No	71	59.7	31	43.7	40	56.3			
**Injecting within 6 months**							–	–	0.22^b^
Yes	2	1.7	2	100.0	0	0.0			
No	116	97.5	54	46.6	62	53.4			
**Ever non injecting drug use**							0.94	1	0.33
Yes	105	88.2	52	49.5	53	50.5			
No	14	11.8	5	35.7	9	64.3			
**Non injecting drug use within 6 months**							4.45	1	**0.04** ^*^
Yes	22	18.5	15	68.2	7	31.8			
No	97	81.5	42	43.3	55	56.7			
**Alcohol Use within 6 months**							–	–	0.19^b^
Yes	5	4.2	4	80.0	1	20.0			
No	114	95.8	53	46.5	61	53.5			
**History of defaulted MMT**							3.41	–	0.20
Nil	79	66.4	39	49.4	40	50.6			
Once	28	23.5	10	35.7	18	64.3			
More than once	12	10.1	8	66.7	4	33.3			
**Ever perform Hep C screening test**							1.42^a^	–	0.51^a^
Yes	103	86.6	51	49.5	52	50.5			
No	10	8.4	3	30.0	7	70.0			
Not sure	6	5.0	3	50.0	3	50.0			
**Hep C screening last year**							0.20	–	0.90
Yes	74	62.2	35	47.3	39	52.7			
No	33	27.7	17	51.5	16	48.5			
Not sure	11	9.2	5	45.5	6	54.5			
**Diagnosed with hep C**							0.64^a^	–	0.78^a^
Yes	38	31.9	20	52.6	18	47.4			
No	75	63.0	34	45.3	41	54.7			
Not sure	6	5.0	3	50.0	3	50.0			
Yes	3	2.5	2	66.7	1	33.3			
No	111	93.3	53	47.7	58	52.3			
Not sure	5	4.2	2	40.0	3	60.0			
**Ever attend Hep C health education program**							4.68	–	0.10
Yes	27	22.7	8	29.6	19	70.4			
No	81	68.1	43	53.1	38	46.9			
Not sure	11	9.2	6	54.5	5	45.5			
**No of Hep C education program attended**							8.76^a^	–	**0.03** ^a^ ^,^ ^*^
Never	86	72.3	47	54.7	39	45.3			
Once	19	16.0	4	21.1	15	78.9			
Twice	5	4.2	1	20.0	4	80.0			
More than 2 times	9	7.6	5	55.6	4	44.4			

Note: (

^a^)- Fisher-Freeman-Halton Exact Test for variables with more than 20% expected cell count less than 5, (

^b^)- Fisher’s Exact Test for variables with more than 20% expected cell count less than 5, (

*) **–** significant p-value less than 0.05.

### Outcome characteristic at baseline

Table 6 shows the study outcome characteristic of the intervention and control group at baseline. There were no significant differences between intervention and control groups in all the outcome variables at baseline.

**Table 6:** Baseline comparison of outcomes between intervention and control group (n = 119).

Outcome construct	Full Sample (n)	Full Sample (%)	Intervention group			Control group			Mann Whitney U test
			n	Median	IQR	n	Median	IQR	p-value
Intention for hepatitis C treatment mean score (T1)	119	100	57	6.50	1.50	62	6.25	1.56	0.851
Total knowledge score (T1)	119	100	57	8.00^a^	4.48^b^	62	8.65^a^	4.06^b^	0.411^c^

Note: (

^a^) – Mean, (

^b^) – Standard deviation, (

^c^)- result of Independent T-Test (Levine’s test p-value > 0.05, equal variance assume was used).

### Effectiveness of HEAL module in increasing intention for hepatitis C treatment across time points (baseline, immediately post intervention and 3-month post intervention) adjusted with covariates

Table 7 described the effectiveness of HEAL module in increasing intention for hepatitis C treatment adjusted for covariates. There was no significant different in intention for hepatitis C treatment between the two groups (β = 0.02, 95% CI = -0.43, 0.47, p-value < 0.92). Participants at 3-month post intervention follow up had higher intention for hepatitis C treatment compared to baseline, adjusted for covariates (β = 0.31, 95% CI = 0.04, 0.57, p-value < 0.05). Looking into interaction between time and group, intervention group at immediately post intervention follow up had higher intention for hepatitis C treatment as compared to control group (β = 0.56, 95% CI = 0.12, 1.01, p-value < 0.01). However, there were no significant different in intention for hepatitis C treatment between the two group at 3-month post intervention follow up.

**Table 7:** Effectiveness of HEAL module in increasing intention for hepatitis C treatment across time points (baseline, immediately post intervention and 3-month post intervention) adjusted with covariates.

Variables	Adjusted β-coefficient (95% CI)	p-value
**Intercept**	5.88 (5.33-6.43)	<0.001
**Study Group**		
Control Group (Ref.)	Reference	
Intervention Group	0.02 (-0.43–0.47)	0.924
Time Point		
Baseline (Ref.)	Reference	
Immediately Post-Intervention	0.23 (-0.07-0.53)	0.138
3 Months Post-Intervention	0.31 (0.04-0.57)	**0.024** ^*^
Group x Time Interaction		
Control Group x Baseline (Ref.)	Reference	
Intervention Group x Baseline	0	
Intervention Group x Immediately Post-Intervention	0.56 (0.12-1.01)	**<0.013** ^*^
Intervention Group x 3 Months Post-Intervention	0.36 (-0.11-0.83)	0.136

Note: (

*)- significant p-value <0.05, CI- confidence interval, Ref-reference category, QIC = 367.896, QICC = 359.928, Intention model adjusted with covariates: race, history of non-injecting drug use in 6 months and number of hepatitis C education program attended.

### Effectiveness of HEAL module in increasing knowledge on hepatitis C infection and treatment across time points (baseline, immediately post intervention and 3-month post intervention) adjusted with covariates

Table 8 described the effectiveness of HEAL module in increasing knowledge on hepatitis C infection and treatment adjusted for covariates. There was no significant different in knowledge on hepatitis C infection and treatment between the two groups (β = 0.021, 95% CI = -1.58, 1.60, p-value < 0.99). However, looking into interaction between time and group, intervention group had significant higher knowledge at immediately post intervention (β = 8.85, 95% CI = 7.35, 10.36, p-value < 0.001) and at 3-month post intervention (β = 5.25, 95% CI = 3.76, 6.75, p-value < 0.001) as compared to the control group. However, the knowledge level among the intervention group reduces at 3-month post intervention follow up as compared to knowledge level at immediately post intervention follow up.

**Table 8:** Effectiveness of HEAL module in increasing knowledge on hepatitis C infection and treatment across time points (baseline, immediately post intervention and 3-month post intervention) adjusted with covariates.

Variables	Adjusted β-coefficient (95% CI)	p-value
Intercept	7.60 (4.86-10.34)	<0.001
Study Group		
Control Group (Ref.)	Reference	
Intervention group	0.01 (-1.58-1.60)	0.992
Time Point		
Baseline (Ref.)	Reference	
Immediately Post-Intervention	-0.08 (-0.91-0.75)	0.849
3 Months Post-Intervention	0.15 (-0.87-1.17)	0.769
Group x Time Interaction		
Control Group x Baseline (Ref.)	Reference	
Intervention Group xBaseline	0	
Intervention Group x Immediately Post-Intervention	8.85	**<0.001** ^*^
Intervention Group x 3 Months Post-Intervention	5.25	**<0.001** ^*^

Note: (

*)- significant p-value <0.05, CI- confidence interval, Ref-reference category, QIC = 6584.794, QICC = 6570.527, knowledge model adjusted with covariates: race, history of non-injecting drug use in 6 months and number of hepatitis C education program attended.

## Discussion

Getting treatment upon being diagnosed with hepatitis C is important especially among the marginalize MMT client’s group to prevent the development of serious complication from chronic hepatitis C infection. Therefore, it is important that these at-risk groups be prepared and had high intention for treatment upon diagnosis. This study aims to evaluate a TPB based HEAL module in increasing MMT client’s intention for hepatitis C treatment along with knowledge on hepatitis C infection and treatment across time point (baseline, immediately post intervention and 3-month post intervention).

The findings of this study demonstrate that the HEAL module significantly increased the intention to participate in hepatitis C treatment among MMT clients immediately after the interventions, as compared to the control group. This suggests that the HEAL module, grounded in the Theory of Planned behaviours, is an effective short-term intervention to enhance treatment intention. However, by the 3-month follow-up, the difference in intention between the intervention and control groups was no longer significant, indicating that the effect of the intervention diminished over time. This decline suggests that while the HEAL module effectively influences behaviour-related intention in the short term, its impact may not be sustained without additional reinforcement.

The reduction in treatment intention over time is an important finding that points to the need for ongoing intervention efforts. While the initial boost in intention highlights the potential of HEAL module to address gaps in awareness and motivation for treatment, it is clear that MMT clients may require follow-up support to sustain their engagement with hepatitis C treatment. One possible explanation for the decline in intention is that, without continuous reinforcement, competing priorities or treatment-related challenges may erode the initial gains made through the intervention.

Nevertheless, there is lack of literature that describe on clinical trial that assess the effectiveness of theory-based health education intervention to increase intention for hepatitis C treatment. Most previous study that observes intention or uptake of hepatitis C treatment was in the form of quasi experimental design. One such design by Mukherjee et al [27] found that, the use of health education module in the form of didactic presentation was able to increase treatment interest among MMT clients (p < 0.001). Didactic approach refers to a teaching approach where by information is pass from a tutor to the target population directly and involve multimodal delivery of information along with discussion to ensure correct information delivered and reenforce the delivery of the information [47]. They conclude that integrating a brief but comprehensive education system within harm reduction services may be a low-cost and effective strategy in improving treatment interest and risk behaviours in resource limited setting, but this intervention needs to be paired with strategies that improve social, economic, and political outcomes for PWID. These findings support our findings that intervention in the form of motion video along with group discussion following the video were able to increase the intention for hepatitis C treatment among intervention group at immediately post intervention. However, strategies to retain the level of intention need to be implement along with the intervention to ensure retention and improvement of the outcome. One article suggest that information retention involve giving a clear meaning to information given and to clearly explain why is the information important along with regular practice of the information given [48]. However, this method is true only for the general population and must be further explore in its practicality among MMT clients.

In oppose to our findings, a clinical trial study by Arain et al [37] found no significant different in willingness for treatment between intervention group and control group across timeline. Their intervention package consists of video and didactic power point presentation regarding hepatitis C along with sharing session by peer who completed the hepatitis C treatment. The different in finding between this study and our study might be due to different in number of respondents recruited. This study only recruits a total of 52 respondent into their intervention and control group whereas our study recruited 119 respondents into both arms. Adequate sample size is crucial component in clinical trial [49,50]. Small sample size trial may lead to variability and increase risk of failing to demonstrate the effectiveness of a given intervention when one really is present [49,50]. Similarly Sakpal [51], further described that too low sample size might result in failure to detect significant findings while sample size overestimation might result in detection of clinical significant findings even though the different might not be clinically meaningful. In addition, the different in result might be due to different in sociodemographic characteristic of respondent and different in tools use to deliver intervention and measure of outcomes.

In increasing respondents likelihood of initiating hepatitis C treatment, a cluster randomized control trial in India by Solomon et al [52], demonstrate that, integrating hepatitis C care into HIV services and a combined individuals testing with individual counselling and education session along with a separate group education session was able to increase the probability of treatment initiation. Their respondents in the intervention group were found to be around 10 times more likely to initiate hepatitis C treatment as compared to those in control group who received usual care. As compared to this intervention, our intervention was conducted among small groups. In comparison between group intervention and individuals face to face session, respondent might be more open to discuss and further clarification of their doubt with service provider in an individual’s session. This may be beneficial in further clarify any of their doubt and help to resolve any personal problem in which they were not comfortable to share among group of people. Thus, a single individual session couple with hepatitis C screening might be beneficial to increase intention and retain it for longer time. However, this strategy might require higher resources especially manpower to conduct the individual session. Among other strategies that can be further explore in future studies is to deliver this module at more frequent interval for example during each clinical consultation session and to display the intervention videos at the clinics waiting places.

In term of hepatitis C infection and treatment knowledge, our study found significant interaction between time and group. HEAL module was able to increase knowledge score significantly at immediately post intervention and at 3-month post intervention among the intervention group as compared to the control group. However, the intention score in the intervention group reduces slightly at 3 months post intervention compared to immediately post intervention but remain significant as compared to control group after adjusting for covariates. This imply that the HEAL module is effective in increasing respondents’ knowledge on hepatitis C infection and treatment. The effect of the intervention module on knowledge remains significant but reduce over time.

Similar findings was observed by Mukherjee et al [27], in which their didactic presentation intervention was able to improve the overall hepatitis C knowledge among their MMT clients respondents (p < 0.001). This finding supports that education intervention is effective in increasing hepatitis C knowledge among MMT clients. Furthermore, our intervention module was able to maintain the significant increase in knowledge at 3-month post intervention. This might be due to the mode of delivery of our intervention in the form of video. Several study have shown that the use of video was able stimulate interest of the respondent in focusing on the material being presented along with facilitate acquisition, retention and recall of information presented [53]. However, further study needs to be done to find ways to increase retention of the knowledge gained.

Another quasi-experimental study by Marinho et al [54] shows similar findings. This study delivers a multi-dimensional education module in the form of education video, leaflet and healthcare professional workshops lasted for 6-month with prespecified topic related to hepatitis C. Following their intervention, they found increases in the level of knowledge (p < 0.001) along with the rate of referral to liver specialist (p < 0.001). This study provides the evidence that multimodal interventions is effective in increasing hepatitis C knowledge among the respondents. As compared to this, our study that only uses video supplement by group discussion was also found to be effective in increasing respondents’ knowledge on hepatitis C. However, as compared to the earlier study, our study implementation requires less resource especially in term of time and human resources. This shows that, as compared to multi-dimensional intervention, simpler intervention module can still effectively increase MMT client’s knowledge on hepatitis C infection and treatment. This finding is especially important in setting with low resource but high burden of hepatitis C.

There were some limitations identified in this study. This study does not measure uptake of hepatitis C treatment among the targeted population because the DAA drug treatment program has not fully expended yet at the government health clinics and it was not embedded in the MMT program itself. Currently it was available in limited quota according to state and cater for all population not specifically MMT clients. Thus, future study should focus on uptake of the treatment once the program fully expended. In addition, data collection from the questionnaire was self-administered by respondents. This might contribute to recall bias because some questions such as injecting and non-injecting drug use, duration of MMT treatment requires respondent to recall the answer. Some of the respondent also contribute to non-response bias due to present of sensitive information such as HIV status and recent drug use. Furthermore, some eligible respondent refuse to participate into the study and some was loss to follow up thus might affecting some important response from being captured in the study. In addition, this study only recruits respondents from government health clinics and does not include MMT client from hospital and from private facilities. Thus, the result of this study cannot be generalized to MMT clients from these facilities.

The HEAL module, designed specifically for MMT clients, shows promise in improving hepatitis C treatment participation and knowledge in Malaysia. Its theory-based structure, grounded in the Theory of Planned Bahaviour, makes it suitable for encouraging treatment intention among this high-risk population. Given the high burden of hepatitis C among MMT clients, the module’s short-term effectiveness suggests that it could be integrated into hepatitis C strategies within Malaysia’s MMT programs.

For wider adoption, the HEAL module could be scaled and implemented across MMT clinics throughout the country. To enhance its long-term effectiveness, strategies such as periodic follow-up sessions or embedding the module into regular care practices may help sustain the initial increases in treatment intention and knowledge over time.

Additionally, the HEAL module aligns well with Malaysia’s national strategic plan to eliminate hepatitis C by 2030. As such, it can serve as a prototype for future initiatives aimed at increasing treatment enrolment among MMT clients. With further adaptation to ensure sustained impact, the HEAL module could significantly contribute to Malaysia’s efforts in improving hepatitis C outcome and advancing national public health goals.

## Conclusion

HEAL module was effective in increasing MMT client’s intention for hepatitis C treatment and knowledge on hepatitis C infection and treatment. However, the benefit of the outcome reduces overtime among this population. Thus, future study should look into effective strategies to retain beneficial intervention outcome among this population. Implementation of this module with effective strategies will further increase and allow for the retention of the desired outcome.

## Supporting information

S1 Fig CONSORT flow diagram. (PDF)

S1 File CONSORT check list. (PDF)

S2 File Study protocol. (PDF)

S1 Dataset Supplementary dataset. (CSV)
