# Supplementary material for: Utilizing Theory of Planned Behaviour to increase intention to participate in hepatitis C treatment therapy among Methadone maintenance therapy clients (MMT) in Malaysia: A cluster randomised control trial
Source: PLoS One. 2025 May 22;20(5):e0324718. doi: 10.1371/journal.pone.0324718 (PMC12097622; doi:10.1371/journal.pone.0324718)
Supplement: S1 Fig — (PDF) [file pone.0324718.s001.pdf]

Lorem Ipsum

Lorem ipsum dolor sit amet, consectetur adipiscing elit. Mauris maximus fringilla ligula, in malesuada erat tempor ac. Quisque dapibus posuere turpis, vel aliquam massa vehicula non.

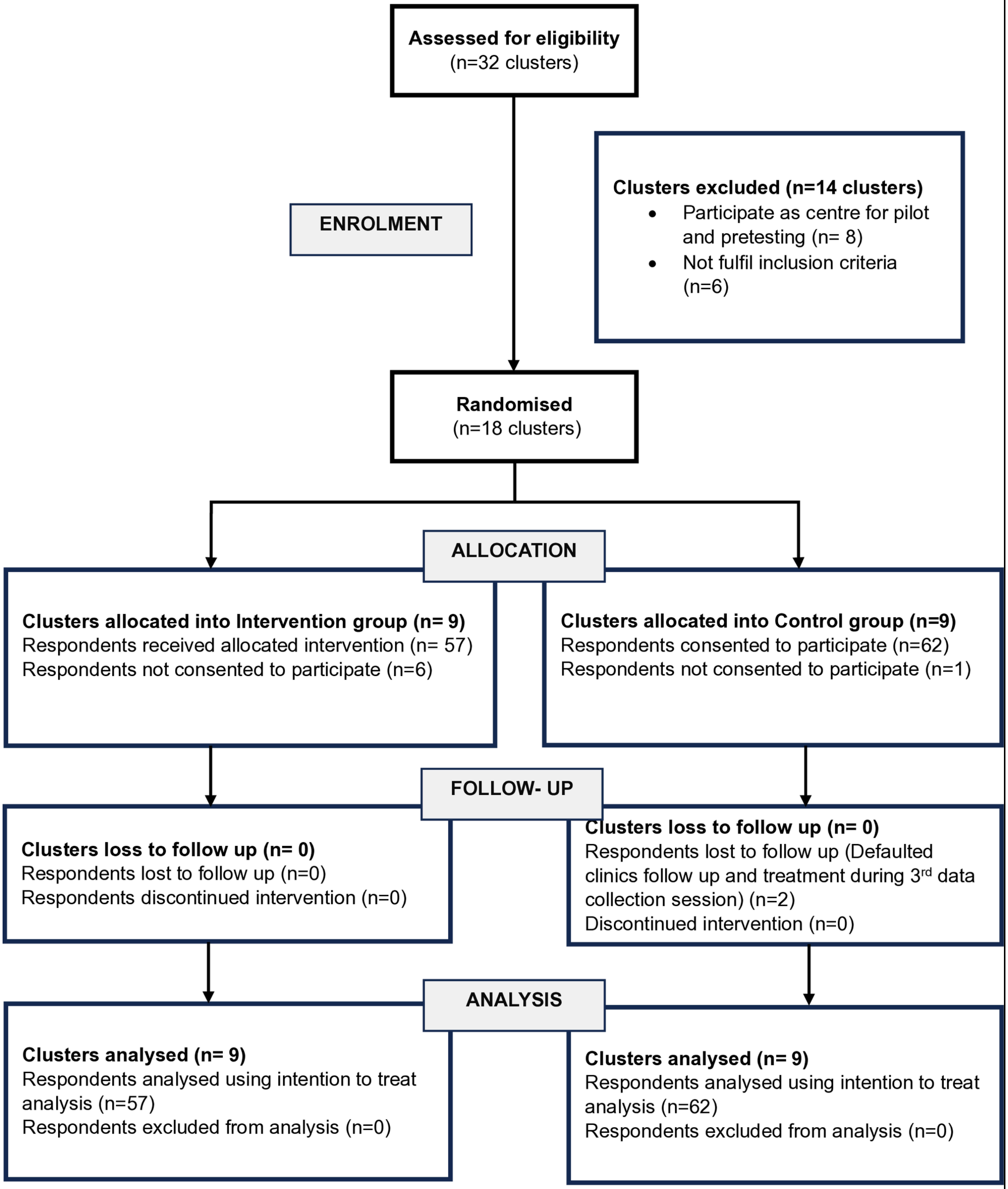

Fig 1. CONSORT flow diagram of the phases of cluster randomize control trial of HEAL module.
