# Supplementary material for: Utilizing Theory of Planned Behaviour to increase intention to participate in hepatitis C treatment therapy among Methadone maintenance therapy clients (MMT) in Malaysia: A cluster randomised control trial
Source: PLoS One. 2025 May 22;20(5):e0324718. doi: 10.1371/journal.pone.0324718 (PMC12097622; doi:10.1371/journal.pone.0324718)
Supplement: S2 File — (PDF) [file pone.0324718.s003.pdf]

STUDY PROTOCOL
VERSION 1.0 DATED 25.10.2022
Study Title:
EFFECTIVENESS OF THEORY OF PLANNNED BEHAVIOR BASED HEPATITIS C EDUCATIONAL INTERVENTION MODULE IN IMPROVING WILLINGNESS FOR HEPATITIS C TREATMENT AMONG METHADONE MAINTENANCE THERAPY CLIENTS IN MALACCA

Prior to commencement of data collection and intervention sessions, ethical approval was obtained from Medical Review and Ethics Committee (MREC), Ministry of health Malaysia (NMRR ID-23-00016-XJF (IIR)) as well as approval to conduct study from Malacca state health department. Furthermore, this study was registered under Thai Clinical Trial Registry (TCTR): TCTR20240504001.

Author : MOHD HAFIIDZ BIN BAHARUDIN

1.1 Background

Malaysia has made a commitment at the 69th World Health Assembly to eliminate hepatitis as major public health threat by 2030 (Ministry of Health [MOH], 2020). To achieve this, 90% infected individuals with hepatitis C need to be diagnosed and 80% hepatitis C infected individual that eligible for treatment will need to be started on treatment by 2030 (WHO, 2017). To show this commitment, Malaysia have taken the initiative to scaled up hepatitis C treatment by making the generic version of DAA available in Malaysia and by launching the Malaysian National strategic plan for hepatitis B and C in 2019. However, it was estimated that in Malaysia, 90% from 380 000 chronic hepatitis C individuals were still undiagnosed and untreated (Mohamed et al., 2019). In addition, the 2018 "Baseline estimates for WHO's 10 core indicators for the Global Reporting System for Hepatitis" have reported that, only 6.1% of hepatitis C patient knew their status and 1.4% were started on treatment. Thus, Malaysia is still far from reaching the targets for hepatitis C elimination set by the WHO.

Malaysia were still in the process of expending the hepatitis C treatment, the generic DAA regimes were only available at selected MOH facilities where limited numbers of patients will be treated in stages (Mohamed et al., 2019). Currently, it was estimate that treatment were only available for around 23000 patient (Mohamed et al., 2019). MMT clients is one of the most important target populations for upscaling hepatitis C screening and treatment. Hepatitis C infections were found to be highly prevalence among MMT clients in Malaysia. The Malaysia methadone treatment outcome study has reported that 64.6% of their respondent among MMT clients were diagnosed with hepatitis C (Ali et al., 2018). In addition, a third of death among MMT clients were found to be related to hepatitis C infection (Ali et al., 2018). Even with the availability of DAA which has high cure rates and more tolerable, it was observed that the awareness and knowledge regarding hepatitis C and the fact that its curable were still very low among MMT clients (Mukherjee et al., 2017 ; Chan et al., 2022). Chan et al., (2022) in their study which is a part of interim review of the Malaysia stratergic plan for hepatitic C further highlight that the reason for hepatitis C treatment were not

widely accepted among people living with hepatitis C was found due to limited disease awareness and disease related stigma. Furthermore, a negative attitude towards hepatitis C treatment could further influence hepatitis C treatment uptake among this population (Yang & Tang, 2000). In addition, some studies have found that more than 40% of MMT clients in their research still engage with illicit drug use along with MMT treatment (L. Li et al., 2012; White et al., 2014). Thus, MMT clients can be considered a pool of individuals at high risk of developing hepatitis C infection due to their history of drug use and current risk of relapse into drug addiction. Moreover, some healthcare workers involve in hepatitis C care on MMT clients find it difficult to convince clients for hepatitis C screening and treatment which reflect the low treatment coverage of 1.4% reported in 2018 (Mukherjee et al., 2017 ; MOH, 2020 ; Chan et al., 2022). This reflect that the willingness of MMT clients to received hepatitis C treatment upon diagnosis were still low and the needs for a program or module to prepare them and increase their willingness for treatment as the government scaled up the hepatitis C treatment in Malaysia.

Currently, there are no comprehensive hepatitis C health education programs in Malaysia to deliver information regarding the advancement in hepatitis C diagnosis and treatment to MMT clients. Furthermore, not much study has been done to investigate the effectiveness of theory-based health education intervention in increasing the willingness for hepatitis C treatment among MMT clients. Hence this study offers to evaluate and add to the body of knowledge on the effectiveness of theory-based health education intervention in increasing MMT client willingness for hepatitis C treatment.

1.2 Objectives

1.2.1 General Objectives

The general objectives of this study are to develop, implement and evaluate the effectiveness of Theory of planned behaviour-based hepatitis C health education intervention module in improving willingness for hepatitis C treatment among MMT clients in Malacca.

1.2.2 Specific Objectives

The specific objectives of this study are:

i. To develop and implement a Theory of planned behaviour-based hepatitis C education intervention module to increase willingness to hepatitis C treatment.
ii. To identify the sociodemographic characteristics, MMT treatment profile, HIV and hepatitis C status, alcohol use, drug history and history of attending hepatitis C educational program of the respondents in the intervention and control group at baseline.
iii. To evaluate the effectiveness of Theory of planned behaviour-based hepatitis C education intervention module in improving willingness for hepatitis C treatment among MMT clients in Malacca.
    i. Within intervention group and within control group from baseline to immediately post-intervention and 3 months post-intervention.
    ii. Between groups (intervention group and control group) at baseline, immediately post-intervention, and 3 months post-intervention.
    iii. Within and between groups over times.
iv. To evaluate the effectiveness of Theory of planned behaviour-based hepatitis C education intervention module in improving knowledge, attitude towards

behaviour, subjective norms, and perceived behavioural control for hepatitis C treatment among MMT clients in Malacca.

i. Within intervention group and within control group from baseline to immediately post-intervention and 3 months post-intervention.
ii. Between groups (intervention group and control group) at baseline, immediately post-intervention, and 3 months post-intervention.
iii. Within and between groups over times.

1.3 Research Hypotheses

The research hypotheses of this study are:

H1- There is higher willingness for hepatitis C treatment within group and between group in intervention group compared to the control group at immediate and 3 months post-intervention.

H2- There is higher knowledge for hepatitis C within group and between group in intervention group compared to the control group at immediate and 3 months post-intervention.

H3- There is higher positive attitude towards hepatitis C infection within group and between group in intervention group compared to the control group at immediate and 3 months post-intervention.

H4- There is higher positive hepatitis C related subjective norms within group and between group in intervention group compared to the control group at immediate and 3 months post-intervention.

H5- There is higher hepatitis C related perceived behavioural control within group and between group in intervention group compared to the control group at immediate and 3 months post-intervention.

METHODOLOGY

1.4 Study Location

This study will be conducted in Malacca which is one of the states in Malaysia.

1.5 Study Duration

This study will commence October 2022 August 2024.

1.6 Study Design

This study is a two-armed, parallel, single blinded, cluster randomized controlled trial (CRCT).

1.7 Sampling

1.7.1 Study Population

The study population recruit for this study will be among the MMT clients registered with the government health clinics facilities from all districts in Malacca.

1.7.2 Sample Population

The sample population will be all registered MMT clients in government health clinics in Malacca attending the MMT clinic from October 2022 until May 2023.

1.7.3 Selection Criteria

1.7.3.1 Inclusion Criteria

The inclusion criteria are:

i. Age 18 years and above
ii. Active MMT clients of government health clinics in Malacca

iii. Negative hepatitis C status or positive hepatitis C status but not on treatment.

1.7.3.2 Exclusion Criteria

The exclusion criteria are:

i. MMT clients diagnosed with hepatitis C and ever or currently on treatment regardless of the sustained virologic response (SVR) status.
ii. MMT clients currently involve in any intervention studies or involved in any intervention study completed within one year before initiation of this study.

1.7.4 Sampling Frame

The list of registration number of MMT clients from the methadone registry obtained from Malacca State Health Department.

1.7.5 Sampling Unit

The sampling unit will be individual who is active MMT clients of government health clinics in Malacca.

Sampling Method

The list of MMT clients based on the registration number were screen for eligibility criteria. Those who does not fulfil the eligibility criteria will be remove from the list. Subsequently, the cleaned registration number list will be arranged in ascending order. Following that each client will be assigned number starting from number one to total number of the population. In the next step simple random sampling will be conducted using online random number generator from https://stattrek.com/statistics/random-number-generator.aspx website to recruit participant according to number of sample size required.

1.7.6 Sample Size Estimation

The sample size calculation for this study was calculated based on the primary outcome desire which is willingness for hepatitis C treatment. The sample size calculation will be calculated using the formula for testing the different in proportion between two groups (Lwanga et al., 1991). The formula and calculation are as depicted below:

$$n = \frac{\{[Z_{(1-\alpha/2)} * \sqrt{2\bar{P}\,(1-\bar{P})}\,] + [Z_{(1-\beta)} * \sqrt{P1(1-P1) + P2(1-P2)}]\}^2}{(P1-P2)^2}$$

$$n = \frac{\{[1.96 * \sqrt{1.19\,(1-0.405)}\,] + [0.842 * \sqrt{0.1875 + 0.2464}]\}^2}{(0.31)^2}$$

n = 39

Thus, the sample size calculated is equal to 39 for one arm. Total sample size for both control and intervention groups are 78 persons (39 x 2). Following that, an additional adjustment for dropouts were made based on the 77% attrition rate from previous study (Arain et al., 2016). Therefore, the adjustment for response rate is 78/(1-L) (L= attrition rate) = 78/0.33= 236 (118 respondents per arm) (KK Gupta et al., 2016). Thus, the sample size needed for this study is 118 pairs of intervention and control groups which amount to 236 subjects. The information used for sample size calculation is as

presented in Table 3.1. The calculation was based on findings from a randomize control trial study for willingness of hepatitis C treatment (Arain et al., 2016).

Table 3.1. Information for sample size calculation

Study Characteristic	Assumption made by investigator
Type of study	Randomize Control Trial
$Z_{(1-\alpha/2)}$	$Z_{(1-\alpha/2)} = 1.96$, the value of the standard normal distribution corresponding to a significance level of alpha of 0.05 for a two-sided test.
$Z_{(1-\beta)}$	$Z_{(1-\beta)} = 0.842$, the value of the standard normal distribution corresponding to the desired level of power of 80%.
P_1	$P_1 = 0.75$, Proportion of respondent willing for treatment in intervention group at 3 months post intervention
P_2	$P_2 = 0.44$, Proportion of respondent willing for treatment in control group at 3 months post intervention
$\bar{P}$	$\bar{P} = (0.75 + 0.44)/2$, $\bar{P} = 0.595$

1.8 Randomisation

1.8.1 Sequence Generation

Upon recruiting the participant who fulfil the eligibility criteria according to sample size calculated, random allocation sequence will be generated to allocate respondents into either intervention or control groups. Randomization sequence will be generated using software from web page “Create a block randomization list” (Sealed Envelope Ltd, 2019) .

In this study, the permuted block randomization will be used to allocate respondent into either the intervention or control group. This permuted block randomization will be conducted using the software from web page “Create a block randomization list” (Sealed Envelope Ltd, 2019). The flow chart of study design will be presented by adopting the CONSORT 2010 flow diagram in the result section after conducting the study as presented in Figure 3.2 (Moher et al., 2010).

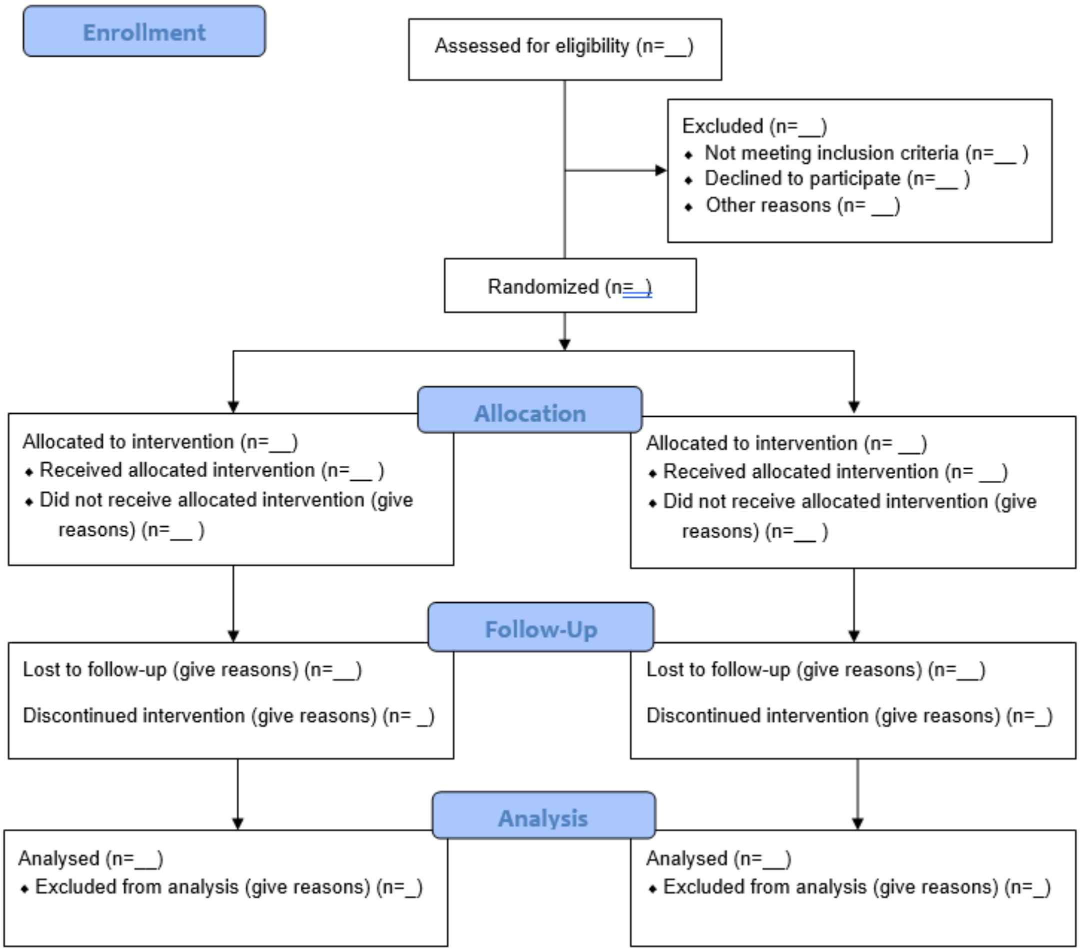

Figure 3.2. Flow Chart of Study Design and Outcome Evaluation

1.8.2 Allocation Concealment

In this study, the allocation concealment method that will be use is the "sequentially numbered, opaque, sealed enveloped methods" as depicted in Figure 3.3. In the process of randomization and allocation concealment, a randomization list will be generated using software from web page "Create a block randomization list" (Sealed Envelope Ltd, 2019). The list will also include unique allocation codes generated by the software which consist of two letters followed by 1 or more digits (E.g: BW2, AM4, AB1 and etc). This unique code will represent either the intervention or the control group and meaning of the code will only be known by the sequence generator. This will ensure concealment of the allocation. Following that, the "sequentially numbered, opaque, sealed enveloped methods" will be use during respondent recruitment. The unique code will be print out, cut out and sealed in opaque envelops. Subsequently, as patient were being recruited, the opaque enveloped containing the different code will be pulled out by liaison officer at each clinic. The randomisation code will only be release after patient have been recruited into the study which take place after the baseline measurement have been completed. By doing this, the parties involve during the randomization process will be blind from the group that the respondent will be allocate

before the unique code being reveal.

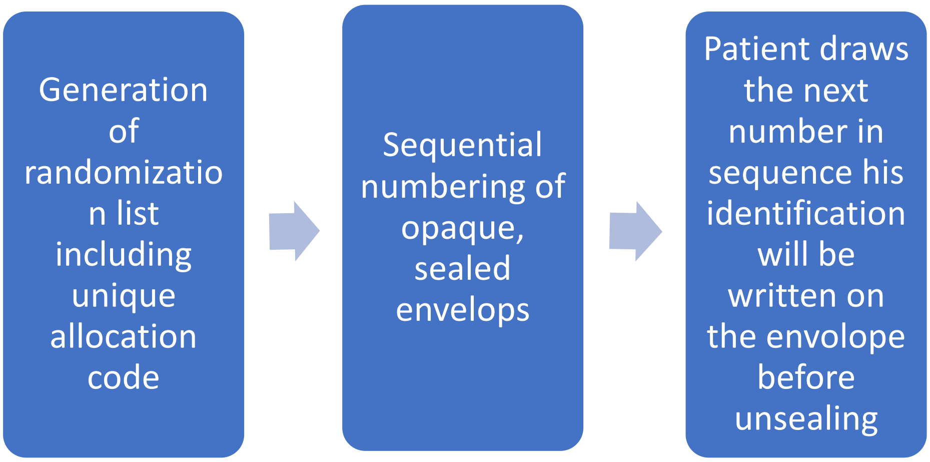

Figure 3.3. Allocation concealment process

1.9 Blinding

This study will use the single blinding method in which the respondent will not be aware of their group status.

1.10 Study Instrument

There are two study instrument that will be use in this study which are health intervention module and questionnaire.

1.10.1 Development of Health Intervention Module

The health intervention module will be developed in Malay language and validated by the supervisory committee and other expert from Universiti Putra Malaysia (UPM) and Malacca State Health Department. The expert will consist of Public Health specialist and Family Medicine Specialist. Figure 3.4 summarize the flow of health education module development.

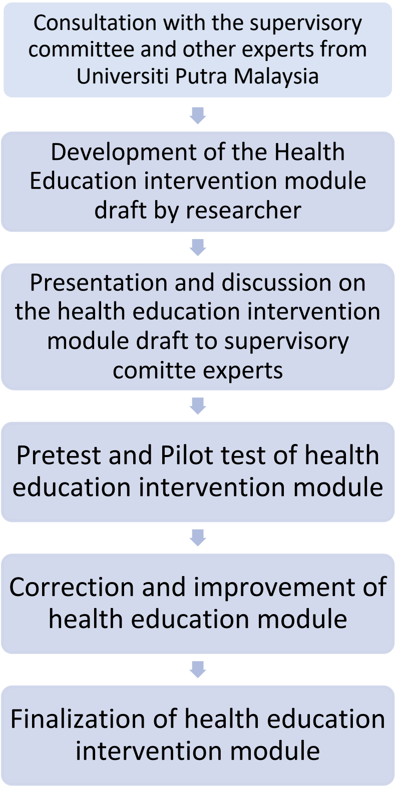

Figure 3.4. The schematic diagram for the development of the health education module on increasing willingness for hepatitis C treatment

The intervention will be delivered during MMT client's appointment with the medical officer in charge of the MMT programs in the form of brief intervention module. The medical officer involve with the study will be brief and undergo training on proper way to conduct questionnaire interview and deliver health education intervention module to the clients. Prior to the intervention session, a baseline assessment will be conducted using validated questionnaire to measure their knowledge regarding hepatitis C, attitude towards hepatitis C infection, subjective norm on hepatitis C treatment, perceived behavioural control on hepatitis C treatment and willingness for hepatitis C treatment. Following that, the brief intervention will be deliver face to face by the medical officer in charge using several materials provided by researcher. The estimated duration of the intervention session is around one and half hours. Table 3.2 summarized the application of TPB constructs in the development of the health education intervention module.

Table 3.2. Content of Intervention Module based on construct of TPB

Construct of theory	Content	Method of delivery	Duration and frequency
Knowledge regarding hepatitis C<br>And<br>Attitude toward behaviour	• Hepatitis C virus, transmission, sign and symptoms, diagnosis.<br>• Risk of hepatitis C, its complication and relation to cirrhosis and hepatocellular carcinoma<br>• Introduction to DAA treatment and comparing to the old interferon treatment	Health talk<br>Video	Content-30 minutes<br>Discussion –10 minutes
Subjective norm on hepatitis C treatment	Experience sharing of patient that successfully completed the Hepatitis C treatment using DAA regime	Recorded video of patient that successfully completed the Hepatitis C treatment using DAA regime and health talk	Content-15 minutes<br>Discussion –10 minutes
Perceived behavioural control on hepatitis C treatment	The barriers to hepatitis C treatment	Recorded video of patient that successfully completed the Hepatitis C treatment using	Content-15 minutes<br>Discussion –10 minutes

		DAA regime and health talk	

Materials which include health talk and pre-recorded video will be prepared and distribute to the medical officer involve in the study.

1.10.2 Questionnaire

Questionnaire that will be use is a self-administered questionnaire that will be distributed to respondent in Malay language. Estimated duration to answer the questionnaire is 20 minutes. The questionnaire section related to knowledge of hepatitis C and construct of TPB will be adopted from previous study. The validity and reliability assessment of the questionnaire will be discussed in respective sections. The set of questionnaires for this study will contain

i. Section A for Sociodemographic characteristics
This section will ask regarding respondent sociodemographic characteristics which include age, gender, race, marital status, employment status, monthly income, and education level.

ii. Section B for MMT treatment profile
This section will explore regarding history of MMT treatment which include current Methadone dose in mg/day and duration of MMT treatment in years.

iii. Section C for HIV and Hepatitis C status
This section will explore regarding respondents HIV and hepatitis C screening history and current HIV and hepatitis C status.

iv. Section D for history of hepatitis C educational program
This section will explore regarding respondents' history of attending any hepatitis C educational program.

v. Section E for substance use history
This section will explore regarding history of smoking and history of ever and past 6 month injecting and non-injecting drug use as well as alcohol use history among the respondents.

vi. Section F for willingness for hepatitis C treatment
This section will explore regarding respondent willingness for hepatitis C treatment is they were diagnosed with hepatitis C and offered for treatment. Respondent will need to select between yes or no to indicate their willingness for hepatitis C treatment.

vii. Section G for knowledge of hepatitis C
This section will explore regarding respondent knowledge on hepatitis C infection, transmission, prevention, and treatment. The questionnaire in this section will be adopted from previous study by Arain et al., (2016). Total item in this scale are 19 items. The answer options will be "True", "False" and "Not sure". Each answer will be given 1 mark for "yes" answer and 0 for "no" and "don't know" answer. Total score will range from 0 to 19 and the higher the score reflect higher knowledge of respondent on hepatitis C infection.

viii. Section H for attitude towards hepatitis C infection and treatment
This section will explore regarding respondent attitude on hepatitis C infection. The questionnaire in this section will be adopted from previous study (Chen et al., 2013). Total item in this scale are 10 items. For first question of this section, 5-point important scale will be apply in which respondent will need to choose from Not important to very important option. For question 2 until 10, the level of agreement of respondent will be rated using 5-point Likert scale ranging from 1 as "strongly disagree" to 5 as "strongly agree". For this section, the total score will

range from 10 to 50. The higher the score indicate the more positive attitude towards hepatitis C infection.

ix. Section I for subjective norm

This section explores regarding clients perceived social pressure on willingness to hepatitis C treatment. This section will be consist of 4 items and the items will be adopted from guidelines on TPB questionnaire (Francis et al., 2004). The level of agreement of respondent will be rated using 7-point scale ranging from 1 as "strongly disagree" to 7 as "strongly agree". For this section, the total score will range from 4 to 28. The higher the score indicate the more positive subjective norm on willingness for hepatitis C treatment.

x. Section J for perceived behavioural control

This section explores regarding clients perceived behavioural control on willingness to hepatitis C treatment. It explore on perceived ease or difficulty of performing the behaviour and it is assumed to reflect past experience as well as anticipated impediments and obstacles (Ajzen, 1991). This section will be consist of 4 items and the items will be adopted from guidelines on TPB questionnaire (Francis et al., 2004). The level of agreement of respondent will be rated using 7-point scale ranging from 1 as "strongly disagree" to 7 as "strongly agree". For this section, the total score will range from 4 to 28. The higher the score indicate the more positive perceived behavioral control on willingness for hepatitis C treatment.

Table 3.3. Summary on source of questionnaire that will be adopted and adapted from previous study

Section	Source of questionnaire adaption	Number of items
Section D: Knowledge of hepatitis C	(Arain et al., 2016)	19 items
Section E: Attitude towards behaviour	(Chen et al., 2013)	10 items
Section F: Subjective norm	(Francis et al., 2004)	4 items
Section G: Perceived behavioural control	(Francis et al., 2004)	4 items

1.11 Quality Control of the study Instruments

1.11.1 Quality control of the Health Education Intervention Module

The developed health education intervention module will be subject to pretesting prior to the full-scale intervention study. The pretest will be conduct among the MMT client that will be exclude from the main study. The objective of pretesting is to obtain feedback from the respondent about the module, to assess the feasibility in implementing the module, and to improve the newly developed health education module. Feedback such as weather the content of module were easily understood, cultural acceptance, flow of the training, resources required in implementing the module (e.g.: time, equipment's, suitability of the person delivering the material, budget), hiccups in implementing, intervention adherence will be assessed to improve the health intervention module.

In addition, the health education module will also be subject to assessment by healthcare provider using the Patient Education Materials Assessment Tool (PEMAT) (Shoemaker et al., 2014). This method is a systematic method to evaluate and compare the understandability and actionability of patient's education material with aim to produce a high-quality material to patient.

After correction of the module based on feedback form pretest assessment, the module will be implemented in the pilot testing, which is small-scale, preliminary study which aim to investigate whether the health intervention module is feasible before conducting in a larger scale interventional study.

1.11.2 Quality control of the Questionnaire

All scale that will be use in this study were adopted from previous study conducted in different countries. Thus, the scale will be translated from English language to Malay language based on the report of the ISPOR Task Force for Translation and Cultural Adaptation (TCA) (Wild et al., 2005). First, forward translation from English to Malay language will be conducted by two translator who were Malay native speaker and proficient in English language. Subsequently, both translators will synthesis the Malay translated version of the questionnaire. Next the two scale will be compared and undergo reconciliation in which any discrepancies will be resolved to obtain a single forward translated version of the scale. Following that, another two translator who have not seen the original questionnaire will be invited to back translated the forward translated version of the scale into original language which is English. Next, the back translated version of questionnaire will be review against the original questionnaire to ensure conceptual equivalence of the translation. Next is the harmonization step in which all version of translation will be compare with the original version of questionnaire to detect and deal with any translation discrepancies that arise between different language versions, thus ensuring conceptual equivalence between the source and target language versions and between all translations.

Subsequently, the translated version of questionnaire will be test in the pretest study to perform the cognitive debriefing of the questionnaire with the aims to assess the level of comprehensibility and cognitive equivalence of the translation, to test any translation alternatives that have not been resolved by the translators and to highlight any items that may be inappropriate at a conceptual level. After correction and finalizing the scale, pilot study will be carried out before the full-scale intervention took place.

1.11.2.1 Face Validity

Face validity of the instrument was performed to evaluate the appropriateness of the phrasing and understanding of the items. It was conducted among two lecturers and three students of the Doctor of public health of Universiti Putra Malaysia (UPM). Subsequently, based on the comments and feedback for improvement by the individuals, the needed modification will be considered.

1.11.2.2 Content Validity

The content validity of questionnaire will be assessed by a group expert. This evaluation will be performed by six experts, which include three public health physicians and three family medicine physicians. The content validation ratio (CVR) will then calculated using formula from a previous study (Lawshe, 1975). Items with a value lower than 0.99 were either eliminated or modified based on the importance of

the items. Furthermore, the comments and feedback by the panel of experts will be taken into consideration for further correction of the questionnaire.

In addition, the Content validity index (CVI) will be calculated to assess the relevance and clarity of the questionnaire by the assigned expert. Following that, the Item CVI (I-CVI) and the scale average CVI (S-CVI/Ave) will be calculated. Polit et al., (2007) recommended a value of 0.78 or higher for I-CVI and 0.90 or higher for S-CVI/Ave to judge a scale as having excellent content validity. The questionnaire will then be modified according to the agreement level of the expert based on the calculated CVI.

1.11.2.3 Reliability

The reliability of the instrument will be assessed using internal consistency, calculated in IBM SPSS (Version 28). Sample size for the reliability test will be determined using an online calculator (https://wnarifin.github.io/ssc_web.html), and MMT clients will be recruited accordingly. These participants will not be included in the main study.

Cronbach's alpha will be used for Likert-scale items, while Kuder-Richardson 20 will be used for dichotomous items. Both methods are recognised as valid for assessing internal consistency (Capik & Gozum, 2015). While a Cronbach's alpha value of $\geq$ 0.70 is commonly accepted as satisfactory, some literature allows values as low as 0.45 (Taber, 2018). For Kuder-Richardson 20, a value of 0.50 or higher is considered acceptable.

1.11.2.4 Training of the Enumerators

Training of the enumerators will be conducted prior to commencement of the intervention. A medical officer in charged for Methadone program from each selected clinics will be appointed as the liaison officer in charge of data collection and distribution of the intervention packages (health talk and video). They will also be responsible for consent form distribution and collection. They will be train on how to conduct the health talk and response to enquiry regarding content of the intervention module.

1.12 Implementation of the Health Education Intervention

An intervention module based on the Theory of Planned Behaviour (TPB) will be delivered to the intervention group through a 20 to 30 minute face-to-face session during routine MMT clinic appointments. Conducted by trained medical officers, the intervention will take place over four months and involve randomly selected MMT clients from government clinics in Malacca.

The session includes three parts: a health talk to improve knowledge and attitudes about hepatitis C; a pre-recorded video of a patient who completed DAA treatment, aimed at enhancing subjective norms; and another video addressing common barriers to treatment.

Before implementation, medical staff will undergo training on delivering the module and conducting interviews. Participants will complete a baseline survey, followed by assessments immediately after the intervention and three months later. The control group will receive the same intervention after the final data collection.

1.13 Data collection process

At the start of the study, clients were randomly selected from the sampling frame and allocated into intervention and control groups. A liaison officer was appointed from each clinic with selected participants and was trained on the study procedures. Eligible clients were approached for consent, and baseline data were collected from those who agreed to participate.

Participants in the intervention group received an intervention package within one to two weeks. The session lasted approximately 30 minutes and included a health talk on hepatitis C and a video featuring a patient sharing their experience with DAA treatment. Outcomes were assessed immediately after the intervention (T1) and again three months later (T2). The control group completed the same assessments at baseline, one to two weeks after baseline (T1), and three months after T1 (T2). After the final assessment, the same intervention package was provided to the control group.

1.14 Study Variables

1.14.1 Dependent Variables

1) Primary outcome

The primary outcome of this study will measure the willingness of the clients for hepatitis C treatment following diagnosis of hepatitis C

2) Secondary outcome

The secondary outcome of the study will be the score of knowledge on hepatitis C, the score of attitudes towards hepatitis C infection, the score of subjective norms and the score on perceived behavioural control

1.14.2 Independent Variables

<u>Hepatitis C Health education intervention</u>

This is the exposure variable of interest in this study. The module will be delivered to the intervention group during the study and to the control group at the end of the study (wait list) if the intervention we found to be effective.

1.14.3 Other Factors Associated with the Primary and Secondary Outcomes

i. Sociodemographic
   a. Age
   b. Gender
   c. Race
   d. Marital Status
   e. Employment status
   f. Monthly income
   g. Education level

ii. Duration of MMT

iii. Alcohol and drug use history

iv. HIV status

v. Hepatitis C status

vi. History of attending Hepatitis C program

1.15 Data Analysis

1.15.1 Statistical Test in Data Analysis

Data collected will be analyse using IBM SPSS (Version 27.0). Data exploration will be performed to look for any error in data entry, missing data and present of outliers.

The distribution of numerical data will then be determined whether they are normally or not-normally distributed. In determining the normality of data both statistical and graphical method will be use. For statistical method, the Skewness/SE of skewness and the Kolmogorov-Smirnov parameter will be calculated, while for the graphical method, the histogram together with stem and leaf box plot will be plotted and analyses. Furthermore, after determining thew normality of data, mean and standard deviation will use to described normally distributed data, while median with first and third quartile (Q1, Q3) will be used to described not normally distributed data. Categorical data will be presented as frequency and percentage.

For between group comparison, the independent sample t-test will be used to compare the mean different of continuous and normally distributed data between intervention and control group. For continuous but not normally distributed data, the Mann-Whitney U test will be used to compare between intervention and control group. The Chi-square test will be used to compare frequency different of categorical data between intervention and control groups and for data of 2 by 2 table that contains cell with an expected count less than 5 for more than 20%, the Fisher's exact test will be use.

For within group changes overtime comparison, the One-way repeated measure ANOVA will be used for continuous and normally distributed variables. Within group analysis for categorical data will use the Cochran's Q test.

The Generalized Linear Mixed Model (GLMM) will be used to test the main effect and interaction between and within the intervention and control groups overtime (baseline, immediately post intervention, and 3-month post intervention). This study will use a significance level with a *p*-value of 0.05 and a confidence interval of 95% for hypothesis testing. For results yielded *p*-value of less than 0.05 will be consider as statistically significant findings.

1.15.2 Strategy of Data Analysis

This study will adopt the intention to treat analysis strategy during data analysis. Intention to treat analysis is a method for analysing results in a randomized study where all participants who are randomized are included in the statistical analysis and analysed according to the group they were originally assigned, regardless of what treatment they received.

In handling missing data, the type of missing data will be first identified whether it is missing completely at random (MCAR), missing at random (MAR) or missing not at random (MNAR). Several techniques exist in handling missing data such as listwise or case deletion, pairwise deletion, mean substitution, regression imputation, last observe case carried forward, maximum likelihood, expectation-maximization (EM) and multiple imputation.

1.16 Ethics Consideration

1.16.1 Ethical Approval for conducting study

Prior to commencing data collection of the study, ethical approval and permission will be requested and obtained from Medical Review and Ethics Committee (MREC), Ministry of health Malaysia. Following that, a written permission to conduct the study in government health clinic in Malacca will be obtained from the Director of Malacca state health department. On top of that, written inform consent will be obtain from each individual respondent before participating in the study (voluntary participation).

Upon MREC approval, the Ethics Committee for Research Involving Human Subject Universit Putra Malaysia (*Jawatankuasa Etika Untuk Penyelidikan Melibatkan Manusia Universiti Putra Malaysia -JKEUPM*) will notify.

1.16.2 Confidentiality

During the questionnaire answering session, the identification of the questionnaire will be conducted by assigning code number, no individual name or any other official identification of respondent will be written in the questionnaire. The intervention and questionnaire answering session will conducted at separate suitable place away from the MMT counter to maintain confidentiality of respondents. The answered questionnaire will be kept in an envelope which was labelled as "confidential" in locked file cabinet by by the assigned site interviewer and will be hand over to the investigator at an assigned date. The confidentiality of the information gain will be maintained, and the identification information will be kept anonymous throughout data analysis.

1.16.3 Duration and means of storage and archival of medical records and study data

Questionnaire will be kept in a secure locked file cabinet with locking mechanism and only principal investigator will have the key to the locker and have access to the questionnaire. The data will enter into SPSS software and access to the data will be encrypted with password to ensure safety and confidentiality of the data. Only the principal investigator will have access to the data. The study data will be kept for 5 years after final publication and only been use particularly for the current study. After the period of storage, the written questionnaires will be shredded, and the electronic data will be deleted.

1.16.4 Respondent access to the personal information and study data

Respondent will not be given access to the personal information and study data. The personal information and study data will be kept confidential and only the principal investigator will have access to the information and data. The data will be analyzed collectively and not by individually.

1.16.5 Publication policy and confidentiality of subject's personal information

All patient details and information obtained from the study will be kept confidential and use only for the research purposes. No identifying information will be recorded in the questionnaire. Each questionnaire will be assigned unique code number and a separate electronic sheet containing respondent name list matched with the unique code number will be kept securely in a password protected laptop and accessible only to the principal investigator. No personal information of the subject respondent will be published. Permission from the Director General of Health, Malaysia, or relevant authorities will be obtained prior to publication. Future publication will be based on the collective data analysis not individual data analysis. The respondent can request the published findings by referring to the final published article on the research.

REFERENCES

Ali, N., Aziz, S., Nordin, S., Mi, N. C., Abdullah, N., Mahmud, M., Paranthaman, V., & Mutalip, M. H. A. (2018). Malaysian Methadone treatment outcome study (MyTOS): Review Malaysian Methadone treatment outcome study (MyTOS): *Ministry of Health, Malaysia*, *16*(March). https://www.researchgate.net/publication/323691778_MALAYSIAN_METHADONE_TREATMENT_OUTCOME_STUDY_Mytos_REVIEW_OF_METHADONE_MAINTENANCE_THERAPY_IN_MALAYSIA_2005-2014/download

Arain, A., De Sousa, J., Corten, K., Verrando, R., Thijs, H., Mathei, C., Buntinx, F., & Robaeys, G. (2016). Pilot study: Combining formal and peer education with fibroscan to increase HCV screening and treatment in persons who use drugs. *Journal of Substance Abuse Treatment*, *67*, 44–49. https://doi.org/10.1016/j.jsat.2016.04.001

Chan, H.-K., Hassali, M. A., Mohammed, N. S., Azlan, A., & Hassan, M. R. A. (2022). Barriers to scaling up hepatitis C treatment in Malaysia: a qualitative study with key stakeholders. *BMC Public Health*, *22*(1), 371. https://doi.org/10.1186/s12889-022-12786-w

Gupta, KK, Attri, J. P., Singh, A., Kaur, H., & Kaur, G. (2016). Basic concepts for sample size calculation: Critical step for any clinical trials! *Saudi Journal of Anaesthesia*, *10*(3), 328–331. https://doi.org/10.4103/1658-354X.174918

Li, L., Lin, C., Wan, D., Zhang, L., & Lai, W. (2012). Concurrent heroin use among methadone maintenance clients in China. *Addictive Behaviors*, *37*(3), 264–268. https://doi.org/10.1016/j.addbeh.2011.11.004

MOH. (2020). *Case Study: Towards Elimination of viral Hepatitis In Malaysia Through Multisectoral Collaboration 2019*. *1*(10), 9–20.

Mohamed, R., Shabaruddin, F. H., Azzeri, A., McDonald, S. A., & Dahlui, M. (2019). Hepatitis C elimination by 2030 in Malaysia: an achievable goal? *Journal of Virus Eradication*, *5*(4), 253. https://pubmed.ncbi.nlm.nih.gov/31754450

Moher, D., Hopewell, S., Schulz, K. F., Montori, V., Gotzsche, P. C., Devereaux, P. J., Elbourne, D., Egger, M., & Altman, D. G. (2010). CONSORT 2010 Explanation and Elaboration: updated guidelines for reporting parallel group randomised trials. *BMJ*, *340*(mar23 1), c869–c869. https://doi.org/10.1136/bmj.c869

Mukherjee, T. I., Pillai, V., Ali, S. H., Altice, F. L., Kamarulzaman, A., & Wickersham, J. A. (2017). Evaluation of a hepatitis C education intervention with clients enrolled in methadone maintenance and needle/syringe programs in Malaysia. *International Journal of Drug Policy*, *47*(203), 144–152. https://doi.org/10.1016/j.drugpo.2017.05.041

White, W. L., Campbell, M. D., Spencer, R. D., Hoffman, H. A., Crissman, B., & DuPont, R. L. (2014). Patterns of abstinence or continued drug use among Methadone maintenance patients and their relation to treatment retention. *Journal of Psychoactive Drugs*, *46*(2), 114–122. https://doi.org/10.1080/02791072.2014.901587

WHO. (2017). *Global hepatitis report, 2017*.

Yang, C., & Tang, D. (2000). Patient-specific carotid plaque progression simulation. *Cmes-Computer Modeling in Engineering & Sciences*, *1*(2), 119–131. https://doi.org/10.1016/j.biotechadv.2011.08.021.Secreted

APPENDICES

APPENDIX A: QUESTIONAIRE

Section A: Sociodemographic

You are required to answer each question listed. For multiple answer questions, please select one (1) best answer by ticking (√).

No	Item	Answer
1	Age	____________ year (Please Specify)
2	Gender	☐ Male<br>☐ Female
3	Race	☐ Malay<br>☐ Chinese<br>☐ Indian<br>☐ Others__________ (Please Specify)
4	Marital Status	☐ Single<br>☐ Married<br>☐ Split up (Not living together)<br>☐ Divorced
5	Employment Status	☐ Have permanent job<br>☐ No permanent job<br>☐ Not working
6	Monthly Income	RM____________ (Please state)

7	Education level	☐	No formal education
		☐	Primary school
		☐	Secondary school
		☐	University/College/Institution (Tertiary)

Section B: Methadone Treatment Profile

You are required to answer each question listed.

No	Item	Answer
1	Current Methadone Dose	____________mg/day
2	Duration from start of Methadone treatment	Year of starting Methadone treatment: __________

Section C: HIV and Hepatitis C status

You are required to answer each question listed. Please answer the following statements regarding HIV and hepatitis C testing and status. Please tick (√) your answer in the space provided.

No	Items	Answer		
		Yes	No	Not Sure
1	Have you ever tested for hepatitis C?			
2	Have you test for hepatitis C last year?			
3	Do you have hepatitis C?			
4	Have you ever tested for HIV?			
5	Have you test for HIV last year? (Only for those without HIV)			
6	Do you have HIV?			

Section D: History of hepatitis C educational program

Please determine the following statements on hepatitis C educational program. Please tick (√) your answer in the space provided.

No	Items	Answer		
		Yes	No	Not Sure
1	Have you ever participated in any form of hepatitis C educational program?			

Section E: Drug and alcohol use history

Please determine the following statements on drug and alcohol use. Please tick (√) your answer in the space provided.

<u>Drug Use</u>

No	Items	Answer	
		Yes	No
1	Have you ever used injecting drug?		
2	If yes, do you have history of injecting drugs use during the last 6 months?		
3	Have you ever used non-injecting drug?		
4	If yes, do you have history of non-injecting drugs use during the last 6 months?		

<u>Alcohol Use</u>

No	Items	Answer	
		Yes	No
1	Have you ever consumed alcohol in the past 1 year? (If yes, proceed to question 2 until 5)		
2	Have you ever felt you should cut down on your drinking?		
3	Have people annoyed you by criticizing your drinking?		
4	Have you ever felt bad or guilty about your drinking?		

5	Have you ever had a drink first thing in the morning to steady your nerves or to get rid of a hangover (eye-opener)?		

Section F: Knowledge on hepatitis C

Please determine whether the following statements hepatitis C infection are true, false or not sure. Please tick (√) your answer in the space provided.

No	Statements	Answer options		
		True	False	Not Sure
1	Hepatitis C is caused by a virus			
2	Hepatitis C is spread by sharing needles for drugs			
3	Hepatitis C is mainly spread by unprotected sex			
4	A person can get hepatitis C by getting a tattoo or piercing			
5	A person can get hepatitis C by sharing personal material like razors or toothbrushes			
6	To be certain of a hepatitis C infection, a blood test is necessary			
7	Hepatitis C damages the liver and can cause liver failure			
8	Hepatitis C can lead to liver cancer			
9	Some people can live many years without symptoms			
10	Part of the people infected with the hepatitis C virus can cure spontaneously			
11	Drinking a lot of alcohol is a good idea for someone with hepatitis C			
12	There is a vaccine to prevent hepatitis C			
13	The treatment for hepatitis C cures everyone who is treated			
14	The treatment for hepatitis C currently consists of injections and taking pills			
15	The treatment for hepatitis C has to be taken lifelong			
16	The treatment for hepatitis C can cause side effects like depression			

17	Substitution treatment can be followed during hepatitis C treatment			
18	Once you completed a treatment for hepatitis C, a reinfection is impossible because you're immune			
19	If you have hepatitis C it is not necessary to get a vaccination for hepatitis A or B			

Section G: Attitude towards hepatitis C infection and treatment

Please indicate your level of agreement with each of the statements below by selecting and marking (√) at your answer choice in the space provided based on the provided scale.

Question 1: Based on Important scale below

No	Statement	Not Important	Slightly Important	Moderately Important	Important	Very Important
1	If you were diagnosed with hepatitis C, how important is it for you to get treatment for hepatitis C?					

Question 2-10: For question 2 to 10, the scale as follow:

1	2	3	4	5
Strongly disagree	Disagree	Neutral	Agree	Strongly Agree

No	Statement	1	2	3	4	5
2	'I do not think that I need to take medication for my hepatitis C'.					
3	'I don't believe that I will die from hepatitis C'.					
4	'Treating my other illnesses is more important than treating my hepatitis C'.					

5	'Treating hepatitis C will make my life better'.					
6	'Hepatitis C does not cause any problems in my body'.					
7	I do not think I need treatment for hepatitis C because I can't tell that I have it' (Asymptomatic)					
8	'Feeling ashamed of having hepatitis C makes me uncomfortable seeking hepatitis C treatment or care'.					
9	'People may treat me differently if they know that I have hepatitis C'.					
10	'I am scared about what might happen to me because I have hepatitis C'.					

Section H: Subjective norms

Please indicate your level of agreement with each of the statements below by **selecting** and **circling** at your answer choice in the space provided based on the provided scale.

1. Most people who are important to me think that...

I Should	1	2	3	4	5	6	7	I should not

…get treatment for hepatitis C if I was diagnosed with it.

2. If I was diagnosed with hepatitis C, it is expected of me that I agree for hepatitis C treatment at the health facilities.

Strongly disagree	1	2	3	4	5	6	7	Strongly agree

3. It is expected for me to be under social pressure to get hepatitis C treatment, if I was diagnosed with hepatitis C.

Strongly disagree	1	2	3	4	5	6	7	Strongly agree

4. People who are important to me want me to go for hepatitis C treatment, if I was diagnosed with hepatitis C.

Strongly disagree	1	2	3	4	5	6	7	Strongly agree

Section I: Perceived behavioural control

Please indicate your level of agreement with each of the statements below by **selecting** and **circling** at your answer choice in the space provided based on the provided scale.

1. I am confident that I can agree on hepatitis C treatment, if I was diagnosed to have hepatitis C.

Strongly disagree	1	2	3	4	5	6	7	Strongly agree

2. For me to agree and go for hepatitis C treatment upon diagnosis is...

Easy	1	2	3	4	5	6	7	Difficult

3. The decision to agree and go for hepatitis C treatment upon diagnosis is beyond my control.

Strongly disagree	1	2	3	4	5	6	7	Strongly agree

4. Whether I agree and go for hepatitis C treatment upon diagnosis is entirely up to me

Strongly disagree	1	2	3	4	5	6	7	Strongly agree

Section J: Willingness for hepatitis C treatment

Based on the statements below, **circle** your answer of choice in the space provided

Question	Answer	
Suppose you were diagnosed to have hepatitis C and was advised for hepatitis C treatment by the medical officer, are you willing to received hepatitis C treatment as advised?	**Yes**	**No**

THE END
